# Fast Detector/First Responder: Interactions between the Superior Colliculus-Pulvinar Pathway and Stimuli Relevant to Primates

**DOI:** 10.3389/fnins.2017.00067

**Published:** 2017-02-17

**Authors:** Sandra C. Soares, Rafael S. Maior, Lynne A. Isbell, Carlos Tomaz, Hisao Nishijo

**Affiliations:** ^1^Department of Education and Psychology, CINTESIS.UA, University of AveiroAveiro, Portugal; ^2^Division of Psychology, Department of Clinical Neuroscience, Karolinska InstituteStockholm, Sweden; ^3^William James Research Center, Instituto Superior de Psicologia AplicadaLisbon, Portugal; ^4^Department of Physiological Sciences, Primate Center, Institute of Biology, University of BrasíliaBrasília, Brazil; ^5^Department of Anthropology, University of California, DavisDavis, CA, USA; ^6^Ceuma University, Neuroscience Research CoordinationSão Luis, Brazil; ^7^System Emotional Science, Graduate School of Medicine and Pharmaceutical Sciences, University of ToyamaToyama, Japan

**Keywords:** superior colliculus, pulvinar, snake detection theory, faces, primates, evolution

## Abstract

Primates are distinguished from other mammals by their heavy reliance on the visual sense, which occurred as a result of natural selection continually favoring those individuals whose visual systems were more responsive to challenges in the natural world. Here we describe two independent but also interrelated visual systems, one cortical and the other subcortical, both of which have been modified and expanded in primates for different functions. Available evidence suggests that while the cortical visual system mainly functions to give primates the ability to assess and adjust to fluid social and ecological environments, the subcortical visual system appears to function as a rapid detector and first responder when time is of the essence, i.e., when survival requires very quick action. We focus here on the subcortical visual system with a review of behavioral and neurophysiological evidence that demonstrates its sensitivity to particular, often emotionally charged, ecological and social stimuli, i.e., snakes and fearful and aggressive facial expressions in conspecifics. We also review the literature on subcortical involvement during another, less emotional, situation that requires rapid detection and response—visually guided reaching and grasping during locomotion—to further emphasize our argument that the subcortical visual system evolved as a rapid detector/first responder, a function that remains in place today. Finally, we argue that investigating deficits in this subcortical system may provide greater understanding of Parkinson's disease and Autism Spectrum disorders (ASD).

## Introduction

Primates are known for their excellent vision, which is often exemplified by statements about their high visual acuity and trichromatic color vision, characteristics shared with no other mammals (Kay and Kirk, 2000; Ross, 2000; Kirk and Kay, 2004; Jacobs, 2008, 2009). High visual acuity is possible because of the presence of a fovea in the retina (Ross, 2000; Kirk and Kay, 2004), and trichromatic color vision, partly because of the presence of genes that produce different kinds of opsin proteins in the retina (Surridge et al., 2003; Jacobs, 2008, 2009), but importantly, not all primates have a fovea or trichromatic color vision (Stone and Johnston, 1981; Ross, 2000; Kirk and Kay, 2004; Jacobs, 2009). What is really special about primate vision is what goes on in the brain after the retina. Neurons from the retina project to two major brain structures, the lateral geniculate nucleus (LGN) and the superior colliculus (SC) (Kaas and Huerta, 1988). From the LGN, signals are sent to the primary visual cortex (V1) and then to other visual areas in the brain (Kaas and Huerta, 1988; Henry and Vidyasagar, 1991; Kaas, 2004). This pathway may be thought of as part of the cortical visual system. From the SC, signals are sent to the pulvinar (PUL), another subcortical nucleus, and thus this pathway may be thought of as part of the subcortical visual system (Kaas and Huerta, 1988). Some signals from the SC are also sent to distinct layers of the LGN (Casagrande, 1994; Preuss, 2007), and in this way the two visual systems, while able to function independently, are also interconnected to some extent fairly early on in visual processing.

The cortical visual system is indeed expansive in primates, especially in anthropoid primates (monkeys and apes) (Barton, 1998; Kaas, 2013). The cortical visual system has been extensively studied and we will not review it here other than to point out that its functions appear to be different from those of the subcortical visual system. Among other functions, the cortical visual system assists the fovea in providing high visual acuity, and integrates form, color, and movement, for example (Hubel and Livingstone, 1987; Kaas and Huerta, 1988; Tanaka et al., 1991; Kobatake and Tanaka, 1994), to help individuals identify objects and to evaluate potential responses to stimuli in their environments.

Given the low proportion of retinal ganglion cells that project to the SC (around 10% in monkeys; Perry and Cowey, 1984), the subcortical system has traditionally been regarded as residual (e.g., Henry and Vidyasagar, 1991). Evidence that has been building slowly over the years is revealing otherwise, however. A subcortical pathway for object recognition is certainly not unique to primates: correlates to the SC and the PUL in non-primate and non-mammal species generally comprise the tectal-thalamic system, which is involved in predator-prey recognition (Ewert, 1970; Sewards and Sewards, 2002). Nevertheless, the great expansion of visual cortical areas and geniculate layers in primates has generally reduced interest in investigating subcortical structures for processing complex visual stimuli. Here we review behavioral and neurophysiological evidence which suggests that the subcortical visual system evolved as a rapid detector of, and first responder to, stimuli that, for individuals relying on the slower cortical visual system, would have dire consequences. We concentrate on snakes and emotional faces of conspecifics as particularly important and well-studied stimuli.

Snakes have been deadly to primates since primates originated, and, indeed, they are argued to have been so important in the evolutionary history of primates that they were largely responsible for the origin of primates via selection on individuals to visually detect snakes before the strike (Isbell, 2006, 2009). One of the hallmarks of being a primate is an expanded visual sense (Cartmill, 1974, 1992), but snakes can also be extremely difficult to see even with excellent vision, and any advantage that helps in their detection should still be favored today. Since constricting snakes have been predators of primates from the beginning of the primate lineage, and venomous snakes are deadly even today for the largest primates if not seen in time, it is also understandable that primates would fear them. Thus, the ability to detect snakes and the fear of them might be linked. Some studies have measured cortisol, a hormone associated with stress and fear, in primates exposed to snakes and have reported elevated levels (Wiener and Levine, 1992; Levine et al., 1993). It is also important to note, however, that fear of snakes in primates may not be inextricably tied to initial detection of and first response to snakes, even though non-human primates typically react strongly to snakes, including visual detection and focused attention, and sometimes mobbing (Seyfarth et al., 1980; Gursky, 2005; Isbell and Etting, 2017) and ophidiophobia is the most common phobia among humans (Agras et al., 1969; APA, 2013). In fact, the relationship between snakes and primates is more nuanced than the snake predator-primate prey relationship suggests. Primates themselves have also long been predators, and competitors, of snakes (Headland and Greene, 2011).

The selective pressure to “read” expressions on faces likely occurred sometime after the initial pressure from snakes. Early primates are thought to have lived as solitary foragers as many small non-primate mammals do today (Gebo, 2004), and thus would have been less social than most of today's primates. Today most primates live in social groups, have flexible facial expressions, and interact frequently with conspecifics over many years (Burrows, 2008; Dobson, 2009; Dobson and Sherwood, 2011). The ability of individuals to detect and respond quickly to a conspecific that intends to do harm, or that sees a dangerous snake or other predator, should be highly advantageous to survival. Detection of and response to angry or fearful conspecific faces may be accompanied by high emotionality even moreso than with the complicated relationship between primates and snakes.

A third aspect in the lives of primates that has not been associated with fear or the subcortical visual system but that nevertheless requires quick detection and response involves visually guided reaching and grasping during locomotion. Primates evolved as arboreal creatures and they are still largely arboreal today (Cartmill, 1974). The locomotor repertoire of many primate species includes frequent, rapid leaps across gaps. In making such leaps, individuals must be able to visually locate quickly, and manually reach for and grasp, particular branches from many meters up in the complicated structure of the forest canopy. Selection against individuals that were not proficient at making such leaps would have been intense. In this review, we provide available evidence, including that from studies of blindsight, that suggests a connection between the subcortical visual system and visually guided reaching and grasping. Thus, the evidence we present argues for the overriding function of the subcortical visual system being that of rapid visual detection and response in life-or-death situations which require such actions, with facilitation of the fear response under certain conditions. We then conclude this review by examining the potential for linkage between deficits in this subcortical system and certain deficits in Parkinson's disease and ASD, two neurological diseases that are not yet fully understood.

## Organization of the SC-PUL

### Superior colliculus

**Figure 1:**
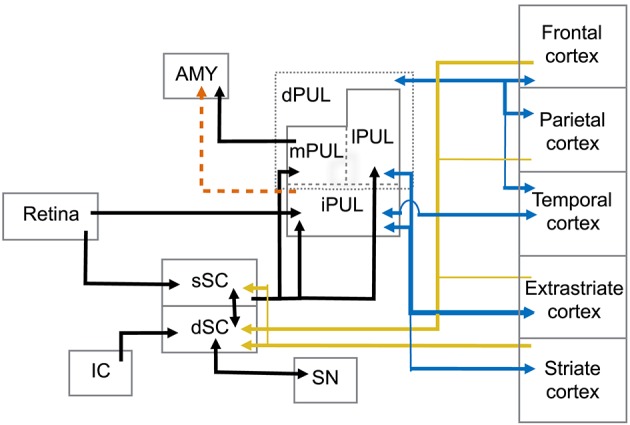
**Abridged summary diagram of the subcortical (SC-PUL) pathway**. sSC, superficial layers of the superior colliculus; dSC, deep layers of the superior colliculus; dPUL, dorsal part of the pulvinar; mPUL, medial pulvinar; lPUL, lateral pulvinar; iPUL, inferior pulvinar; IC, inferior colliculus; SN, substantia nigra; AMY, amygdala. Red dashed line: observed in non-primates (tree-shrew). Blue lines: pulvinar-cortical and cortical-pulvinar connections; gold lines: cortico-collicular connections.

The superior colliculus (SC) is a laminated structure positioned at the tectum of the mesencephalon in the primate brain. Based on its anatomy and functional properties, it is commonly divided into superficial and deep layers of neurons (see May, 2006, for detailed discussion) (Figure 1). The superficial layers of the superior colliculus (sSC) receive direct input from the retina (Leventhal et al., 1981; Perry and Cowey, 1984; Rodieck and Watanabe, 1993). Neurons in sSC have retinotopically organized receptive fields (Lund, 1972; Sparks, 1986). Visual information from sSC reaches both the PUL and LGN in the thalamus (Huerta and Harting, 1983; Stepniewska et al., 2000). Both the PUL and LGN, in turn, maintain reciprocal connections with a number of cortical areas such as V1, V2, and MT (Benevento and Fallon, 1975; Linke et al., 1999; Grieve et al., 2000; Kaas and Lyon, 2007; Schmidt et al., 2010).

The deep layers (dSC) are further subdivided into intermediate and deep zones and both present a more multimodal response profile, in which neurons respond not only to visual, but also acoustic and somatosensory stimulation (Jay and Sparks, 1987; Groh and Sparks, 1996). Neurons in the dSC are also involved in premotor circuits of eye and head movements (Sparks and Mays, 1981; Lee et al., 1997), although eye movements are not required for the dSC's role in attention (Ignashchenkova et al., 2004). Extensive electrophysiological studies with primates, including single-unit recording and electrical stimulation, show that activity in some dSC neurons induces saccadic shifts and head movements (Cowie and Robinson, 1994; Freedman and Sparks, 1997; Ignashchenkova et al., 2004). In this sense, the dSC receives direct cortical input from the frontal eye field (FEF) and supplemental eye field (SEF) areas and lateral intraparietal cortex (LIP). The dSC also projects back to these cortical areas through thalamic relays (Harting et al., 1980). Interestingly, LIP target neurons receive input from the sSC through pulvinar relays and, in turn, project back to dSC layers (Clower et al., 2001). The dSC is also the target of parietal and prefrontal cortical areas involved in the control of purposeful arm/hand movements (Borra et al., 2014), indicating its role in eye-hand coordination (Lünenburger et al., 2001). Auditory information is mapped within the dSC layers where inferior colliculus projections converge with visual representation (Huerta and Harting, 1984; Jay and Sparks, 1987). There are also inhibitory interlaminar connections between the cells in the sSC and dSC with similar receptive fields (Moschovakis et al., 1988), possibly integrating visual and auditory fields.

Activation of the dSC also results in motor responses, particularly defensive behaviors. In rodents, stimulation of the dSC elicits a range of motor responses, including defensive behaviors such as cowering and freezing (Ellard and Goodale, 1988; Brandão et al., 2003). Recently, it has been shown that activation of dSC neurons in macaques by GABAergic antagonism induces similar responses of cowering and escape behavior (DesJardin et al., 2013). These behaviors are likely to rely on connections between the SC and substantia nigra. The nigrotectal pathway has been described (Beckstead and Frankfurter, 1982; Huerta et al., 1991) as well as collicular input to the substantia nigra in a few primate species (May, 2006).

### Pulvinar

A main function of the PUL is to assist in visual processing by shifting attention to relevant stimuli and tuning out irrelevant visual information (Ungerleider and Christensen, 1979; LaBerge and Buchsbaum, 1990; Chalupa, 1991; Robinson and Petersen, 1992; Robinson, 1993; Morris et al., 1997; Grieve et al., 2000; Bender and Youakim, 2001). It is the largest nucleus in the thalamus of primates and is especially large in anthropoid primates (Walker, 1938; Jones, 1985; Chalupa, 1991; Stepniewska, 2004; but see Chalfin et al., 2007). It can be divided into several divisions, including a ventral part (vPUL) comprised of the inferior PUL (iPUL) and ventral portions of the lateral PUL (lPUL) (Stepniewska, 2004; Preuss, 2007; Figure 1). The iPUL and ventral lPUL are visual, receiving inputs from the retina and sSC (Clower et al., 2001) and projecting to many different visual areas including V1, V2, and the superior temporal sulcus (STS; Stepniewska, 2004).

The dorsal part of the PUL (dPUL) is comprised of the dorsal portion of the lPUL and the multisensory medial PUL (mPUL). The dPUL may not exist in non-primates but has greatly expanded in anthropoid primates (Preuss, 2007). Like the vPUL, the dPUL is involved in attention and orientation to salient visual stimuli (Robinson and Petersen, 1992), and its expansion suggests an increased importance of the subcortical processing of relevant stimuli. Importantly, although the dPUL has connections with more cortical areas than the vPUL, it does not have connections to V1 (Trojanowski and Jacobson, 1974; Glendenning et al., 1975; Baleydier and Mauguière, 1985, 1987; Selemon and Goldman-Rakic, 1988; Acuña et al., 1990; Garey et al., 1991; Robinson and Petersen, 1992; Baizer et al., 1993; Ma et al., 1998; Gutierrez et al., 2000; Stepniewska, 2004). Furthermore, the dPUL receives inputs from the dSC (Stepniewska, 2004) and projects to the lateral amygdala (Jones and Burton, 1976; Aggleton and Saunders, 2000).

## The SC-PUL circuit for affective stimuli

A possible pathway from the sSC and dSC to the dPUL that excludes VI but goes to the amygdala points to a way for visual input to reach the amygdala without cortical involvement. Another possible pathway might involve sSC neurons reaching the vPUL, and then being relayed to the dPUL and amygdala. This route has been demonstrated in non-primate mammals (Day-Brown et al., 2010). To our knowledge, however, this route is lacking in tracer studies in the primate brain. A direct SC-PUL connection to the amygdala has recently been predicted in humans and macaques by means of probabilistic diffusion tensor imaging tractography (DTI; Tamietto et al., 2012; Rafal et al., 2015). It is important to note that DTI provides an indirect anatomical finding that will require confirmation from neurophysiological tracing studies, which is still lacking in primates. In either case, a subcortical visual route that sends signals to the amygdala has attracted the attention of several neuroscientists in the past 10 years or so, as it has critical implications for the study of affective stimuli.

Several recording studies have generated supporting evidence for a SC-PUL role in affective/salient visual stimuli. As a whole, the SC receives visual input mostly from magnocellular and koniocellular channels, which yields information with low-spatial resolution (Miller et al., 1980). In theory, the subcortical visual pathway would relay fast and low-detailed visual information (“fast and coarse”) for immediate response. Recordings from SC and PUL neurons in behaving macaques indicate these nuclei encode facial and threatening stimuli significantly faster than the visual cortex, as early as 25 ms and 40 ms, respectively (Maior et al., 2011; Nguyen et al., 2013, 2014; see below). This has also been supported by magnetoencephalographic (MEG) studies with a dynamic causal modeling (DCM) in which a fast subcortical visual pathway yielded more explanatory power for short latency responses compared to a cortical model (Garrido et al., 2012; Garvert et al., 2014). This ascending (feedforward) information may be further amplified by interactive activity based on reciprocal connections between the SC-PUL pathway and cortical areas (Shipp, 2003; Pessoa and Adolphs, 2010). The cortico-PUL-cortical circuits are involved in amplifying signals and improving signal-to-noise ratios (Shipp, 2003; Pessoa and Adolphs, 2010), as well as modulating interactions between oscillatory processes in different cortical areas, which contributes to visual attention (Serences and Yantis, 2006; Saalmann and Kastner, 2009).

Taken together, anatomical, behavioral, and recording findings in primates are consistent with the current (but as we argue here, limited) view that the SC-PUL pathway functions to direct visual attention to salient emotional stimuli. Below we examine evidence for two kinds of stimuli in particular that have almost certainly had profound effects on primate survival over evolutionary time: snakes and emotionally expressive faces.

## The SC-PUL and snakes

### Behavioral evidence

Fear plays a critical role in helping organisms deal with potentially dangerous encounters by being associated with rapid and effective defensive responses (immobility, flight, fight, e.g., Blanchard and Blanchard, 1983). Öhman and Mineka (2001, 2003) proposed that defense systems imposed by vulnerability to snakes over evolutionary time (Isbell, 2006, 2009) shaped the appearance of a “fear module” in their prey—an independent behavioral, psychophysiological, and neural system that is relatively encapsulated from more advanced human cognition. Isbell (2006, 2009) has also emphasized the evolutionary importance of snakes by arguing that natural selection has shaped primates to quickly detect snakes and respond appropriately to them, including responding with fearful behavior. According to the Snake Detection Theory (SDT), the pressure posed by snakes over evolutionary time favored the origin of primates by selecting for visual systems that are highly sensitive to snakes (Isbell, 2006, 2009).

Inspired by evolutionary considerations, long-term research programs from several laboratories and spanning several decades have generated a large body of evidence showing that stimuli involving some level of evolutionarily derived threat, such as potentially dangerous animals, engage different neurobehavioral systems from those evoked by more mundane and innocuous stimuli, thus with preferential access to the fear module (see Öhman and Mineka, 2001). As the result of ancient evolutionary co-existence between snakes and primates, fear of snakes is still highly prevalent in both humans (e.g., Agras et al., 1969; Fredrikson et al., 1996; Lang et al., 1997) and monkeys (Mineka et al., 1980). Furthermore, when snakes are paired with aversive events, fear conditioning is more rapid and stable than to neutral stimuli, again both in humans (e.g., Öhman et al., 1978) and other primates (e.g., Cook and Mineka, 1990), and independently of whether it involves direct or vicarious conditioning (i.e., observing other monkeys displaying fear of snakes).

These fear-relevant stimuli also serve as effective fear stimuli even when masked from conscious recognition (Öhman and Soares, 1993, 1994; Carlsson et al., 2004) and shown under perceptually degraded conditions (Kawai and He, 2016), and are more rapidly detected—i.e., have attentional priority—when presented among distractor stimuli (e.g., flowers, mushrooms) in visual search tasks (e.g., Öhman et al., 2001). This preferential processing has been consistently shown with adult humans (e.g., Öhman et al., 2001) and small children (LoBue and DeLoache, 2008; LoBue et al., 2010; Masataka et al., 2010; Hayakawa et al., 2011; Penkunas and Coss, 2013a,b; Yorzinski et al., 2014), as well as with lab-reared, snake-naïve macaques (Shibasaki and Kawai, 2009). The invariant snake-scale patterns are also highly salient visual cues, as shown by several field studies (e.g., Ramakrishnan et al., 2005; Meno et al., 2013; Isbell and Etting, 2017).

Despite multiple demonstrations that the fear module is selectively sensitive and automatically activated by snakes (see Öhman and Mineka, 2003), the results from most of these studies preclude a direct test of the role of evolution in emotion, since no equivalent animal fear stimuli with distinctive evolutionary histories with primates have been used as a comparison stimulus. For humans, spiders may represent an ideal candidate since they involve matched fear levels to those of snakes—reflected in valence, arousal, and dominance ratings (Lang et al., 2005), and are both highly frequent objects of phobias (e.g., Agras et al., 1969; APA, 2013). However, since non-human primates do not react fearfully to spiders but sometimes perceive them as food items, fear of spiders is undoubtedly younger evolutionarily than fear of snakes. This makes them the ideal comparison stimuli for testing the implications of the SDT (e.g., Steen et al., 2004; Isbell, 2009). Although some studies have indeed included spiders as an evolutionary fear-relevant stimulus, unfortunately, the authors combined them into the same category with snakes, thus impeding the study of any potential dissociations between the two (e.g., Öhman et al., 2001).

When spiders and snakes are separated as experimental stimuli, as a growing body of research has demonstrated, humans show preferential detection of snakes compared to spiders. Soares and her colleagues (e.g., Soares et al., 2014) performed a series of behavioral experiments in humans to test predictions of one of the hypotheses of the SDT, i.e., that the vital need to detect dangerous snakes under challenging visual conditions provided a strong source of selection for the evolution of visual solutions to this threat. The results were supportive, and showed that humans preferentially detected snakes (compared to spiders and mushrooms) under taxing visual conditions, namely when the stimuli were presented more rapidly (Soares and Esteves, 2013; Soares et al., 2014), in the visual periphery (Soares et al., 2014), in a cluttered environment (Soares et al., 2009, 2014; Soares, 2012; Soares and Esteves, 2013), and when attention had to be automatically redirected to suddenly appearing snakes in the immediate environment (Soares, 2012; Soares and Esteves, 2013; Soares et al., 2014). Additionally, a study using an interocular suppression technique—the Continuous Flash Suppression (CFS; Tsuchiya and Koch, 2005), known to suppress stimuli from awareness, showed that snakes overcame suppression and accessed awareness faster than spiders (and compared to birds), again in the most visually demanding conditions—when the stimuli were presented to the participant's non-dominant eye (Gomes et al., 2017).

The dissociations between snake and spider processing were recently extended to non-human primates in a visual search study with snake-naïve Japanese macaques, showing that snakes were detected significantly faster than non-threatening animals (koalas), whereas the detection of spiders did not differ from the innocuous stimuli (Kawai and Koda, 2016). Importantly, and in order to study the attentional time course of the privileged processing of snake stimuli, further recent studies have used event-related potentials (ERPs) and complemented these previous findings by showing that snakes depict earlier visual attention in passive viewing tasks compared to spiders (and innocuous animal stimuli), as reflected in larger early posterior negativity (EPN) amplitudes (He et al., 2014; Van Strien et al., 2014a,b, 2016), with the curvilinear shapes of snakes only partially explaining this enhancement (Van Strien et al., 2016). Finally, a study by Grassini et al. (2016) showed that enhanced EPN amplitudes to snakes were only observed when the stimuli were presented under aware conditions. Although this result contradicts previous findings (see Gomes et al., 2017), the authors relied on different methodologies to manipulate awareness. While Grassini et al. (2016) relied on masking procedures, Gomes et al. (2017) used breaking CFS (b-CFS), which seems to enable suppression from visual awareness for longer periods of time (Lin and He, 2009).

Together, this consistent bulk of data showing a preferential specificity for snake processing invites an evolutionary explanation, such as the one offered by the SDT, while also suggesting that spider fear may be confined to humans and generated more through mechanisms of learning (see Soares et al., 2009).

### Neurophysiological evidence

Based on extensive studies, the SC-PUL pathway was proposed as the “low road” of affective visual stimuli to the amygdala (LeDoux, 1996). Fearful (threatening) images passing through both structures would elicit fast amygdalar activation, which, in turn, would trigger autonomic and behavioral responses. A large number of experiments using threatening social stimuli (e.g., fearful or aggressive facial expressions) on human and non-human primates has corroborated this framework (see below). In contrast, evidence for preferential activity of the SC-PUL visual pathway toward snake stimuli is more limited and comes from a handful of recent studies employing lesion, imaging, and electrophysiological recordings. Although they were not specifically designed to test the predictions of SDT, their results largely support a phylogenetic predisposition for fast snake detection.

Regarding the SC, bilateral neurotoxic lesions in infant capuchin monkeys impaired the emotional processing of snakes as threatening stimuli (Maior et al., 2011). Lesioned monkeys in that experiment were uninhibited by the presence of a rubber snake in a threat-reward task, whereas control monkeys refrained from approaching the food reward next to it, even after 12 h of food deprivation. Although this result is suggestive of SC importance in processing visual threat stimuli, it does not, by itself, hint of any preferential processing of snakes specifically because snakes were not compared with other stimuli. It is interesting to note, however, that central visual field or foveal representations in the SC seem also to be very sensitive to snake images in humans. In a human fMRI study by Almeida et al. (2015), snake stimuli presented in SC regions representing the fovea elicited increased activity. This central sensitivity indicates that the SC is not just engaged during orientation to peripherally presented stimuli.

Snake-sensitive neurons were also found in the PUL of Japanese macaques, particularly in its medial and dorsolaterally portions (Le et al., 2013). In this case, snake stimuli elicited faster and stronger responses from PUL neurons than other stimuli, including emotional faces of conspecifics. Latencies were found to be a little longer than in SC neurons (~55 ms), a finding which is in line with expected for the second relay in the subcortical pathway model. Furthermore, low-pass filtering (LPF) of snake images did not affect neuronal firing, and high-pass filtering (HPF) decreased it, suggesting that PUL neurons process low spatial frequency (LSF) stimuli. In a subsequent study, Le et al. (2014) showed that the PUL might code not only for the presence of threatening stimuli but also for the degree of threat. In that study, a larger subset of PUL neurons was more sensitive to snake pictures depicting striking postures than non-striking postures in that response magnitudes were significantly higher to snakes in striking postures. Furthermore, PUL neurons display gamma oscillation in response to snake images, suggesting feedforward processing for images of snakes, consistent with rapid detection of snakes (Le et al., 2016).

Since the pulvinar is highly interconnected with both cortical areas and subcortical nuclei (Acuña et al., 1990; Baizer et al., 1993; Ma et al., 1998), its functions regarding snake processing may be proportionally varied and complex. Recent findings in humans from Almeida et al. (2015) mirror the results described above. The SC, PUL, and the amygdala displayed differential activation to true snake stimuli (vs. cables, strings, etc.). Interestingly, there was a very strong pattern of fMRI activation to centrally presented snake pictures in all three structures. This central sensitivity suggests again that the SC-PUL pathway is not only engaged in attention to peripherally presented stimuli but is also involved in explicit processing of snakes.

Taken together, the findings of these studies point to particular features of snake stimuli processing: (1) *Short response latencies*: single-cell experiments show faster responses to snakes compared to other threatening stimuli, including expressive faces. SC neurons, on average, fired at slightly shorter latencies than PUL neurons, 20–100 ms and 30–120 ms, respectively. It is possible that extremely short-latency PUL neurons receive direct input from the retina, bypassing SC (Nakagawa and Tanaka, 1984). (2) *Stronger response magnitudes*: PUL neurons showed stronger firing to snakes compared to facial expressions. SC neurons with central and lower visual fields, on the other hand, showed similar response magnitudes to snake and faces; (3) *Spatial frequencies*: PUL neurons are known to be sensitive to LSF images (Schiller et al., 1979; Vuilleumier et al., 2003). Accordingly, PUL neuronal responses were unaffected by LPF of snake images, but were significantly decreased by high spatial frequency (HPF) (Le et al., 2013). At low levels of spatial frequencies, images depict broad features without fine visual details. (4) *Visual field locations*: central visual field areas in the SC seem to be very sensitive to snakes. This suggests that preferential processing of snakes includes early spatial detection as well as explicit central processing. (5) *Naivety*: SC-PUL activity may be independent of previous interactions with snakes, as monkey subjects were often lab-reared and very unlikely to have seen snakes before the experiments. This is particularly striking in the case of behavioral avoidance of a snake model by sham-lesioned monkeys (Maior et al., 2011).

## SC-PUL and faces

### Behavioral evidence

Faces are important means of communicating potential threats to observers. Facial expressions of anger, for instance, signal imminent aggression toward the observer, while faces expressing fear are indicative of potential danger in the environment. Both facial expressions, therefore, signal a possible threat to the individual, albeit with each having particular features in regard to their detection. In this sense, several studies have shown that the threatening nature of these stimuli is maximal when angry faces are coupled with a direct gaze, which is indicative of a threat directed to the observer, and with an averted gaze in fear faces, since it provides the observer with a more precise indication of where the threat is located (e.g., Adams et al., 2003). Because faces expressing anger and fear may jeopardize the protection of the self (Fridlund, 1994), several researchers have proposed that they are part of an evolved response system, together with snakes and perhaps other predatory animals (for a review, see Öhman et al., 2012).

This notion is supported by substantial behavioral data demonstrating that angry and fearful faces are more effectively detected as targets in visual search tasks (e.g., Öhman et al., 2010; Pinkham et al., 2010), are more difficult to ignore, both in humans (e.g., Fox et al., 2001; Georgiou et al., 2005) and in macaques (Landman et al., 2014; Kawai et al., 2016), potentiate perceptual abilities of non-threatening stimuli presented subsequently (e.g., Becker, 2009), enhance perception and attentional capacities (e.g., Phelps et al., 2006; Bocanegra and Zeelenberg, 2012), increase distraction of task-irrelevant items across the visual field and under increased attentional load conditions (e.g., Lavie et al., 2003; Berggren et al., 2013), show faster acquisition and more resistance to extinction in conditioning procedures (e.g., Öhman et al., 1985, 1995), and gain preferential access to awareness (e.g., Yang et al., 2007).

The pattern of behavioral results arguing in favor of a more efficient ability to detect threatening social stimuli is, however, less clear than that observed for snake stimuli. Although considerable data on the effects of angry faces on visual attention argue in favor of an evolutionarily tuned threat detectability, the literature is mixed, with about half the articles in favor of an evolutionarily tuned threat detection system (a so-called anger superiority effect) and the other half showing more efficient detection of happy faces in attracting attention (for an overview, see Lundqvist et al., 2014; see also Becker et al., 2011). The latter evidence, i.e., that happy faces (compared to threatening faces) are more rapidly detected in visual search tasks, seems to be particularly evident when the stimuli depict photographs of real faces and not schematic ones (Becker et al., 2011; Lundqvist et al., 2014), with the most parsimonious account for these results relying on visual conspicuity and not on the emotional nature of the stimuli (e.g., Calvo and Marrero, 2009). Importantly, a recent meta-analysis of several studies exploring the influence of emotional facial stimuli in visual attention suggested that emotional arousal can explain the mixed findings (Lundqvist et al., 2014). Indeed, most of the previous research in this domain assumed the relationship between emotion and attention from a valence and not from an arousal perspective. The findings from Lundqvist and colleagues make evolutionary sense as arousal reflects the degree of energy and mobilization for eventual fight or flight responses (e.g., Lang and Bradley, 2010), which might then be under the purview of the SC-PUL pathway.

Another study with the goal of resolving conflicting findings in the emotion-attention domain advanced gender as an additional factor modulating the anger superiority effect (Öhman et al., 2010). More specifically, angry male faces are more rapidly and accurately detected in a visual search setting than angry female faces, consistent with the view that males are more associated with hostility, and females, with friendliness.

A similar pattern emerges in emotion recognition tasks, where a pervasive happy face advantage (faster reaction times and higher accuracy) is observed across diverse manipulations (for a meta-analysis, see Nummenmaa and Calvo, 2015). However, these studies have mainly relied on prototypical full-intensity expressions. It might well be expected that there is enhanced recognition accuracy for angry faces at lower intensities since the survival premium of efficient recognition of a threatening face when the emotional intensity is subtle may promote adaptive behavioral responses in situations where potentially aggressive encounters are imminent (for a review, see Öhman et al., 2012).

Conditioning to threat faces also seems to be less robust than that observed for snakes. For instance, in one study verbal instructions eliminated the fear-conditioned responses to angry faces (Rowles et al., 2012), while a different study demonstrated that aversively conditioned angry faces only captured attention under low load conditions (Yates et al., 2010). These findings suggest that faces are probably more context-specific than are snakes.

Finally, a recent study showed that although fearful faces gained preferential access to awareness (using CFS), compared to neutral faces, this advantage relied on HPF information (Stein et al., 2014), thus suggesting involvement of cortical visual processing (e.g., Schiller et al., 1979). These results are inconsistent with the role of the retino-collicular-pulvinar-amygdala pathway (see LeDoux, 1996) in this privileged access to awareness. However, Stein and colleagues also open the possibility that biologically relevant stimuli, such as snakes, show an advantage in accessing awareness based on LSF information, which argues in favor of an SC-PUL pathway to the amygdala. This would highlight that social and predatory fear stimuli may have distinctive neuronal signatures, given their different biological relevance (see Öhman et al., 2012). This reasoning conforms to the relative evolutionary importance of snakes and angry or fearful faces, with the former being important from the beginning of the primate lineage when predatory snakes were present but primates are thought to have been limited in their social interactions (see Isbell, 2006, 2009), and the latter emerging later, as primates became more social, with greater fluidity in social interactions requiring more cortical processing to assess and respond to social cues. However, as will be shown in the next section, the relative importance of SC-PUL and cortical vision in assessing emotion from faces may also be dependent on ontogeny.

### Neurophysiological evidence

Several lines of investigation support the claim that faces are a special class of stimuli in the primate visual system (Grüsser and Landis, 1991; Carey, 1992). The human cortex includes dedicated areas for facial stimuli processing, most notably the fusiform face area (FFA) in the lateral fusiform gyrus (Kanwisher et al., 1997). Together with the FFA, the inferior occipital gyrus, posterior superior temporal sulcus, and the anterior infero-temporal cortex have shown differential activation for faces compared to other objects (Rossion et al., 2012). There is, however, mounting evidence that facial information is also processed in a parallel subcortical circuit involving the SC-PUL.

One important line of evidence refers to the preference for faces displayed by human babies (Johnson et al., 1991). Neonates tend to orient their gaze to face-like stimuli immediately after birth (Goren et al., 1975). At this point in development, cortical structures are not fully mature and show only limited activation (Johnson, 2011; Cohen Kadosh et al., 2013). Control of visually guided tasks in newborns is very likely exerted by visually related subcortical structures (Csibra et al., 1998, 2000). Based on these findings, the two-process theory of face processing posits that an innate disposition to faces is supported by subcortical structures in newborns while cortical regions gradually specialize in facial detection and recognition throughout development (Johnson and Morton, 1991; Johnson et al., 2015).

Newborn preference for upright faces is a potentially thorny issue in this field. Such an effect is present immediately after birth but disappears after 2 months, only to reemerge at around 6 months of age (Mondloch et al., 1999; Nakano and Nakatani, 2014). This U-shaped preference for faces called into question the reliability of earlier findings drawn from newborn studies. Nevertheless, a recent study employing S-cone sensitive stimuli (Nakano et al., 2013) has shed light on this controversy as well as underscored the involvement of the SC in facial detection. Since the SC is “blind” to S-cone stimuli, Nakano and colleagues were able to show that 2-month-old babies have a preference for S-cone upright faces. This indicates that the apparent disappearance of this preference for upright facial stimuli may be the result of changes in the hierarchical organization of visual areas in the brain.

S-cone stimuli have also been used to demonstrate the contribution of subcortical structures to rapid detection of faces. In general, faces induce shorter reaction times compared to non-facial neutral stimuli in neuropsychological studies (Crouzet and Thorpe, 2011). Emotional expressions conveyed by faces seem to induce even shorter reaction times as in the case of fearful faces vs. neutral faces. These effects disappear when facial stimuli in S-cone isolating frequencies are used (Nakano et al., 2013), indicating that the facilitatory effect for fast detection relies on collicular activity. Although S-cone isolating results should be viewed with caution (see Hall and Colby, 2013), this finding is further corroborated by Garvert et al. (2014). Using the MEG approach, they compared the dynamic causal models for different latencies of face processing. The authors found that, at least for short latencies, data from evoked fields pointed to a direct subcortical connection to the amygdala for facial stimuli with varying degrees of emotional expressions.

The influence of the SC-PUL pathway in facial detection is also demonstrated by the neuro-ophthalmological syndrome known as “blindsight” (Sanders et al., 1974; Stoerig and Cowey, 1997). In broad terms it refers to the ability to unconsciously detect and discriminate visual stimuli after destruction of striate cortex (“cortical blindness”). Patients with this syndrome are able to accurately guess motion, position, and some aspects of images presented in their blind visual fields (Weiskrantz, 1996). This effect extends to faces (Solca et al., 2015) and emotionally salient stimuli (more narrowly termed as “affective blindsight”; Celeghin et al., 2015). Neuroscientists have taken advantage of this phenomenon to investigate the underlying processes not normally noticed during conscious experience. One patient (G.Y.) with cortical blindness in the right half-field has been shown to recognize different emotional faces (de Gelder et al., 1999). In a later study with the same patient, fearful faces induced differential amygdalar responses that correlated with activity in posterior thalamus and SC (Morris et al., 2001). Moreover, the presentation of fearful faces to the blind hemifield of hemianopic patients enhanced responses to facial stimuli presented to the cortically-intact hemifield (Anders et al., 2009; Cecere et al., 2014). These findings underscore the importance of SC-PUL in processing affective facial stimuli. Interestingly, one recent study raised the possibility that many visual deficits in patients with Parkinson's disease may be due to the inhibition of the SC and dysfunctional PUL activity (see also Isbell, 2009; Diederich et al., 2014).

There are also several techniques to elicit unconscious responses to faces by subliminal presentation of pictures, such as backward/forward masking and continuous flash suppression (see Axelrod et al., 2015, for review). Combined with fMRI techniques, they have provided further evidence of SC-PUL participation. Subliminal fearful faces in fMRI were correlated with activation of a direct subcortical pathway in a feedforward connection (Williams et al., 2006). Troiani and Schultz (2013) found activation in the SC, amygdala, thalamus (PUL), and hippocampus for suppressed objects (including emotional expressions of fear). Interestingly, suppressed faces failed to elicit activation in the FFA, indicating that SC-PUL processing of such stimuli may occur independently, at least in the short term, without cortical input.

Since neurons in the primate SC-PUL pathway are tuned for broad, LSF information, investigators have used low-frequency and high-frequency filtered pictures to selective activate visual channels. Vuilleumier et al. (2003) used low-frequency filtered fearful faces to induce activation of the SC and PUL. This activation was correlated with a stronger amygdalar response compared to high-filtered faces. Low-pass filtered facial (neutral) stimuli subliminally presented produced congruence when guessing the gender of the faces (Khalid et al., 2013). This is indicative that some aspects of faces may be distinctively coded in the SC and PUL. Indeed, in an elegant protocol based on the monocular segregation of visual inputs, Gabay et al. (2014) provided evidence that the subcortical visual pathway conveys representation of identity in facial stimuli.

Studies with macaques have largely supported the findings from human studies while yielding a more detailed profile of SC and PUL neuronal behavior. Sensitivity to face-like patterns was detected in individual SC neurons in macaque monkeys as early as 25 ms (Nguyen et al., 2014). Although actual faces did not elicit differential responses compared to face-like patterns, these neurons also showed differential responses to different gaze direction. Face-like patterns also elicited responses from lPUL and mPUL neurons within 50 ms and between 50 and 100 ms (Nguyen et al., 2013). The activation in the first 50-ms interval was restricted to a few aspects of the stimuli and it is consistent with the activity of SC neurons toward the same kind of stimuli (Nguyen et al., 2014). The activity in the 50-100-ms interval and later, in contrast, was observed in a greater number of neurons and those encoded more information from the stimuli. This later activation of PUL neurons may involve inputs from descending cortical neurons and it is in keeping with an analysis of gamma oscillations in the PUL of macaques (Le et al., 2016). Gamma oscillations are thought to occur simultaneously in areas processing visually attended stimuli. In this particular case, the pulvinar would be involved in cortico-cortical integration for face stimuli processing. Recording of single-unit neurons in the PUL of macaques showed that these neurons respond to different emotional expressions of human faces (Maior et al., 2010). The latency of response in this case varied from ~40 ms to over 300 ms, which is consistent with both a first, fast and coarse feed-forward response, and a later cortical integration.

Altogether, the available data are consistent with the “fast and coarse” scenario for the SC-PUL pathway. Short latency response from SC neurons seems to give an early indication of facial patterns, including orientation, gender, and identity information. PUL neurons seem to be sensitive to the same aspects but they also participate in early cortical processing of facial expressions. The possible targets for SC-PUL facial-related information include several cortical areas and subcortical nuclei but the pathway is likely to provide information for fast amygdalar facial responses at around 100 ms (Tazumi et al., 2010).

## SC-PUL and visually guided reaching, grasping, and pointing

As the previous sections have described, growing evidence suggests that the SC-PUL pathway is a fully functioning system for rapid, non-conscious detection of evolutionarily salient stimuli that often necessitate a rapid response for the responder's continued survival, particularly when dealing with emotionally charged stimuli, i.e., snakes and emotional faces. Some of this knowledge has arisen from investigation of perturbations of the neocortex. Thus, as described above, blindsight, in which the primary visual cortex is non-functioning, reveals the sensitivity of the SC-PUL system in detecting emotional faces (Morris et al., 2001; Vuilleumier et al., 2003).

Growing evidence suggests, however, that we have not yet tapped into all the evolutionarily relevant functions of the SC-PUL system. If the SC-PUL system is well suited as a fast detector/first responder in primates, there is at least one other evolutionarily salient condition that requires a rapid, non-conscious response, and it is observable via studies of blindsight. In addition to affective blindsight, which strongly implicates the SC-PUL's involvement in non-conscious detection of certain types of emotional faces, there is action blindsight, in which the ability to make saccades to targets, and reach for and grasp or, for humans, to also point to targets very quickly, remains (Weiskrantz et al., 1974, 1995; Barbur et al., 1980, 1999; Blythe et al., 1987; Stoerig et al., 1997; Danckert and Rossetti, 2005; Carey et al., 2008). Although the SC-PUL system has been implicated in action blindsight (Danckert and Rossetti, 2005), no evolutionary explanation has yet been offered. Why would it be so important to be able to non-consciously and rapidly reach for and grasp, or point to, objects?

Except for felids and primates, mammals are not known to have visually guided reaching and grasping. Conventionally, this ability has been attributed to visual predation as the earliest primates are thought to have been insectivorous (Cartmill, 1974). However, there is some evidence that visually guided reaching and grasping is not actually universal among primates. Macaques and humans are indeed capable of visually correcting errors in reaching and grasping (Pessiglione et al., 2003; Schettino et al., 2003, 2006; Danckert and Rossetti, 2005). However, despite being highly insectivorous, galagos apparently cannot adjust their arms to grasp a moving target once they initiate the movement (Bishop, 1964).

From a neural point of view, the inability of galagos to use vision to adjust their online reach may be related to their more limited connections between visual areas and regions of the posterior parietal cortex (PPC) that are involved in reaching and grasping (Stepniewska et al., 2005). From an evolutionary point of view, the apparent inability of galagos to adjust their reach with visual feedback may be related to their mode of locomotion. Small non-primate mammals generally move in arboreal habitats by scurrying along the tops of branches, minimizing large leaps across gaps, and using claws to help them grip when necessary. The last common ancestor of all primates is also thought to have been small and arboreal but with nails instead of claws (Gebo, 2004), which would have required a prehensile grip on small branches. Primates now range widely in body size, and have different modes of locomotion with different ways of crossing arboreal gaps: vertical clingers and leapers, such as galagos, leap with their hindlimbs landing first; quadrupedal, above-branch walkers leap with their forelimbs landing first, and; suspensory, below-branch graspers or brachiators use their forelimbs to swing from branch to branch.

We are concerned here with the latter two types, both of which involve forelimb-dominated locomotion. When quadrupedal, above-branch walkers and suspensory, below-branch graspers cross arboreal gaps, they must quickly and accurately with their forelimbs reach for and grasp branches that often differ in orientation and circumference. There would have been strong selection favoring visually guided forelimb reaching and grasping in the ancestors of such primates since missing a target branch just once can be fatal.

De Winter and Oxnard (2001) have shown that locomotor style has influenced the coordinated evolution of certain brain components in primates. Compared with bats and insectivores, primates have expanded several regions of the brain that are involved in voluntary motor control. Furthermore, locomotor styles and correlated expansion of these regions within primates cluster together regardless of phylogenetic relatedness, with scurriers and hind limb-dominated vertical clingers and leapers separated from above-branch leapers, and all separated from suspensory graspers. Humans are outliers among primates, having expanded those same regions of the brain the most (de Winter and Oxnard, 2001). Although, as bipedal walkers and runners, humans no longer need their forelimbs in locomotion, they have developed extensive manual tool manufacture and use, and humans are thought to be the only species that engages in declarative pointing, i.e., the motor behavior of pointing to an object as a way to direct another's attention to it for the purpose of sharing interest in it (Povinelli and Davis, 1994; Butterworth et al., 2002; Tomasello et al., 2007; but see Leavens et al., 2005). Thus, more generally, directed forelimb action may have fueled brain expansion in primates in ways that are different from other mammals.

Here we pull together several indirect lines of evidence to suggest that, in concert with higher cortical systems involved with forelimb-dominated locomotion or forelimb directed action, the SC-PUL system supports rapid, non-conscious, visually guided reaching and grasping, and pointing (see also Isbell, 2009). In the primate neocortex, reaching and grasping, and for humans, pointing, in addition, are heavily represented in the PPC, which is part of the dorsal “vision for action” stream (Previc, 1990; Goodale and Milner, 1992; Goodale and Westwood, 2004). The SC and PUL both contribute to the dorsal stream: the SC sends projections indirectly to the PPC through the PUL (Lyon et al., 2010), and the PUL sends projections directly to the PPC (Selemon and Goldman-Rakic, 1988; Schmahmann and Pandya, 1990).

Stimulation of the SC's deeper layers in vertebrates, including primates, results in bodily movement as well as oculomotor movement (Ewert, 1970; Werner, 1993; Gandhi and Katnani, 2011). Neurons have been found in the SC of macaques and humans that are involved in both oculomotor responses and reaching and grasping (Werner, 1993; Lünenburger et al., 2000; Stuphorn et al., 2000; Nagy et al., 2006), suggesting integration of visual and motor behaviors, which would seem critical in visually guided reaching and grasping. For example, neurons (“gaze-related reach neurons”) fire in the SC when arm movements reach for targets in the direction of the gaze, and arm movements also speed up saccades (“saccade neurons”) to those targets (Lünenburger et al., 2000; Stuphorn et al., 2000; Snyder et al., 2002). As another example, “fixation” neurons in the SC allow primates to visually lock onto a target once it has been located (Krauzlis et al., 2000), and arm movements modulate these fixation neurons (Lünenburger et al., 2001). Finally, neurons in the SC have been found to anchor the gaze of a person to any target to which that person points (Stuphorn et al., 2000; Neggers and Bekkering, 2002).

In macaques, PUL neurons have been found to respond more strongly to visually guided, intentional reaching movements to targets than to passive or exploratory arm movements (Margariños-Ascone et al., 1988; Acuña et al., 1990), whereas neurons in the PPC respond more to passive arm movements (Acuña et al., 1990). In addition, firing rates of PUL neurons are more strongly correlated with rapid arm movements than with force (Margariños-Ascone et al., 1988). A minority of PUL neurons also respond more quickly than neurons in the PPC (Acuña et al., 1990), suggesting that bottom-up processing might occur.

Individual neurons have also been found in the PUL that are responsive to both visual stimuli and movements of the arms and hands, again suggesting integration of visuo-motor abilities (Martín-Rodriguez et al., 1982; Margariños-Ascone et al., 1988). In an fMRI study of humans, the PUL was activated when visual and hand movements occurred together but not when either occurred alone (Ellerman et al., 1998). In macaques, temporary inactivation of the dPUL results in a poorer ability to reach for and grasp objects (Wilke et al., 2010).

Available evidence thus suggests that the SC-PUL system functions more broadly than orienting attention to salient emotional stimuli. It may be more accurate to describe the SC-PUL system as a first detector of and responder to stimuli that require rapid visual detection and motor responses for continued survival. In this regard, the SC-PUL system in non-human primates appears to be tripartite in having heightened sensitivity to (1) snakes as a potential threat, (2) emotionally charged social cues, i.e., emotional faces, and (3) graspable objects in the environment. It has further been suggested that in humans, declarative pointing was built on two of the three functions of the SC-PUL system, having evolved initially from visually guided reaching and grasping and later as a social response that improved avoidance of snakes (Isbell, 2009). Since blindsight studies also demonstrate that the ability to point to “unseen” targets still remains, the evidence thus far suggests that the SC-PUL system participates in the processing of declarative pointing.

By recognizing that the SC-PUL system also plays a role in motor responses for visually guided reaching and grasping, and pointing (in humans), we can expand our current understanding of it and view it as an integrated visual/motor system that is required to detect and respond very quickly, and thus, for maximum benefit, non-consciously.

## Future explorations

The combined results of multiple studies corroborate the notion that the SC-PUL pathway forwards “rapid and coarse” visual information about snakes. Indeed, both the SC and PUL fire faster or more strongly for snakes compared to faces (neutral and fearful), snake-like objects, hand, and simple pattern objects. Although facial expressions may signal social and non-social threat, the remaining comparison stimuli are neutral with regard to threat. The SDT, and the involvement of the SC-PUL pathway, is therefore strongly supported from a neurophysiological perspective. Nevertheless, it is worth stressing the limited spectrum of stimuli employed so far. Thus, studies that compare SC-PUL responses to snakes relative to other threatening stimuli, especially natural predators such as felids or raptors, would be informative. This approach would further clarify the role of the SC-PUL in threat detection, innate object recognition, and the SDT. In addition, future studies comparing behavioral and neurophysiological processing of these different types of stimuli in aware and unaware conditions (e.g., by using interocular suppression techniques), would provide new insights on the heated debate regarding the automatic nature of fear stimuli processing. With regard to rapid detection of emotionally charged faces, future studies could investigate whether emotional intensity is a critical factor in search efficiency in detection tasks. Finally, comparative studies are invaluable for understanding the current role of cerebral regions. In general, neuroscientific studies have been performed on a handful of primate species, most of which are closely related macaques. By generalizing results from such few species, our field is nearly blind to ecological and evolutionary clues to the origin and function of brain systems. This seems particularly critical in the case of the SC-PUL system as it is so intimately related to critical survival responses.

### The SC-PUL and Parkinson's disease

Another way to explore the functions of the SC-PUL pathway might be to investigate whether Parkinson's disease (PD) adversely affects the patient's ability to detect emotional faces, to detect and respond appropriately to snakes (and perhaps other biological threats), to reach for and grasp objects, and to point declaratively (Isbell, 2009; Diederich et al., 2014). PD adversely affects the SC-PUL system beginning with the retina and the SC via loss of dopamine from the substantia nigra (Djamgoz et al., 1997; Dommett et al., 2005; Armstrong, 2011). It also damages the PUL and the amygdala (Harding et al., 2002; Diederich et al., 2014).

PD sufferers are indeed less sensitive than non-sufferers to emotional facial expressions (Sprengelmeyer et al., 2003; Armstrong, 2011), perhaps because they are also less sensitive to contrast at lower spatial frequencies (Davidsdottir et al., 2005; Hipp et al., 2014), the frequency realm of the SC-PUL system. As mentioned above, neurons in the SC and PUL are highly sensitive to images of emotional faces (as well as snakes) at low spatial frequencies (Vuilleumier et al., 2003; Le et al., 2013).

Parkinson's patients also have deficits in reaching and grasping (Klockgether and Dichgans, 1994; Lu et al., 2010). For example, PD sufferers who cannot see their hands when they point to or grasp a target can miss the target (Klockgether and Dichgans, 1994). They are often also slower than unaffected people to shape the fingers to grasp, and their shaping movements become even slower without visual feedback (Schettino et al., 2003, 2006; Ansuini et al., 2010). With the automaticity of the dorsal stream, including the SC-PUL circuit, impaired, the burden to adjust is then placed on non-automatic visual and cognitive processes, which may become overloaded, thus causing even greater dysfunction (Lu et al., 2010; Pieruccini-Faria et al., 2014; Nemanich and Earhart, 2016).

While we are unaware of any studies that have deliberately tested PD patients for their responses to snakes or other biologically relevant threats, we note that PD patients often “freeze” as they approach a doorway or an object in their path (Azulay et al., 2006; Okuma, 2006; Cowie et al., 2010; Snijders et al., 2010). Under natural conditions, abrupt freezing is a normal response to rapid visual detection of threatening stimuli, including peripheral and looming objects and dangerous objects in one's path. The SC-PUL visual system is responsive to such stimuli (e.g., looming objects; Billington et al., 2011). Stimulation of the deeper layers of the SC also causes animals to freeze and lesions of the deeper layers abolish defensive behavior (Ellard and Goodale, 1988; Northmore et al., 1988; Sewards and Sewards, 2002; Brandão et al., 2003; DesJardin et al., 2013).

Freezing in PD patients is frequently associated with visual deficits in contrast sensitivity at lower spatial frequencies (Davidsdottir et al., 2005) and in response and speed of saccades (Nemanich and Earhart, 2016), suggesting SC-PUL system impairment. Thus, some of the locomotor deficits in PD might reflect impaired visual detection and an over-response to potential danger.

Future studies might consider investigating the possible role of the SC-PUL visual system in rapid visual detection/rapid motor responses (e.g., freezing) in primates, particularly with regard to snakes and other predators, reaching and grasping, and, in humans, pointing. One promising approach might be to involve patients with PD to test the hypothesis that some of their visual and motor deficits are influenced by damage to the SC-PUL pathway. If our interpretation is correct that freezing is a response to the SC-PUL's danger detection function, with PD the response would then be an over-response whereas the response to emotional facial expressions is an under-response. Testing is needed, however, because it is unclear why these responses would be different.

### The SC-PUL and autism spectrum disorders

Several lines of evidence suggest that the SC-PUL pathway might also be involved in the pathology of autism. First, ASD are defined by deficits in social reciprocity and communication, and by unusually restricted, repetitive behaviors (American Psychiatric Association, 2000). Social deficits may be critical to identifying autism's etiology (Schultz, 2005). As reviewed above, SC-PUL neurons respond well to facial photos and face-like patterns (Nguyen et al., 2013, 2014), and population activity of SC-PUL neurons discriminates facial identity, gender, and face orientation in the early latencies (before 100 ms after stimulus onset) (Nguyen et al., 2017). Faces provide important information for triggering social behaviors, and coarse (LSF) information is important for face recognition in newborn babies with relatively immature visual cortical areas (Johnson, 2005; de Heering et al., 2008). Recent studies indicate that holistic face perception is largely supported by low spatial frequencies and suggest that holistic processing precedes the analysis of local features during face perception (Goffaux and Rossion, 2006), and face contours (similar to the face-like patterns in the SC-PUL neurophysiological studies) shortened response latencies to faces in the human occipito-temporal regions (Shibata et al., 2002). This evidence suggests that the SC-PUL pathway plays an important role in social behaviors in early infants before they develop the cortical system for full social behaviors, and that social deficits in autism might be ascribed to some deficits in the SC-PUL system. Consistent with this hypothesis is the finding that declarative pointing, a social behavior that normally develops by about 12 months of age (Tomasello, 2000; Liszkowski et al., 2004), is not done by children with autism (Mundy et al., 1986; Baron-Cohen, 1989, 1995). Moreover, an fMRI study reported that activity in the SC-PUL pathway was substantially reduced in patients with autism in response to facial photos (Kleinhans et al., 2011). A neurophysiological study analyzing evoked potentials also reported that autistic children showed a bias toward HSF stimuli (fearful face, gratings) compared with LSF stimuli, in contrast to control subjects, again suggesting that the subcortical visual pathway including the SC-PUL might be affected in autism (Vlamings et al., 2010). Finally, lesions of the SC induced transient decreases in social behaviors in infant monkeys (Maior et al., 2012).

Second, several studies suggest deficits in disengagement of visual attention as a unique feature of autism in young children (Rodier, 2000; Landry and Bryson, 2004; Elsabbagh et al., 2009, 2013). Orienting attention to a new target requires three sequential mental operations: (1) disengagement of attention from its current focus; (2) moving attention to the new target; and (3) engagement of the new target (Posner et al., 1984; Posner and Petersen, 1990). These studies investigated orienting reactions of young children with and without autism who looked at 3 computer monitors in front of them. Once attention was engaged on a fixation stimulus in the central monitor, a second stimulus was presented on either side, either simultaneously (overlap condition) or successively (gap condition). Reaction time to the peripheral stimuli (new targets) was longer in those children with autism in the overlap condition, in which disengagement of attention to the central monitor was required. Deficits in disengagement are one of the earliest symptoms observed in the development of this disorder and such deficits may underlie the social and cognitive impairments observed in patients with autism (Keehn et al., 2013; Sacrey et al., 2014). The idea that the SC might be involved in attention disengagement processes, and SC malfunctioning and/or malformation might be related to the origin and development of autism, was tested in a behavioral study in which rats were trained in a light-guided spatial choice task (de Araujo et al., 2015). At each trial, the rats had to choose one of two paths, leading either to a large or a small reward, based on cue light(s). In this task, the same cue light (frequent cue light) was repeatedly presented, and another cue light (infrequent cue light) was sometimes presented simultaneously with the frequent cue light. The rats could acquire a large reward if they chose the infrequent cue light, in which both attentional disengagement and shift of attention from the frequent cue light were required. The study indicated that temporary inactivation of the SC selectively impaired performance in this task. A neurophysiological study in rats supports these findings in demonstrating the existence of SC neurons that are related to attention disengagement as well as attention engagement in a comparative task (Ngan et al., 2015). These neurons showed excitatory responses during presentation of a cue light contralateral to the recording sites if the cue required attentional disengagement from an ipsilateral cue light. Furthermore, behavioral latencies to the contralateral cue light requiring attentional disengagement were negatively correlated with response magnitudes of the disengagement-related neurons to the contralateral cue light requiring attentional disengagement. Consistent with these results, a human case study reported that a patient with lesions including the right SC showed deficits in saccades to the contralateral (left) target in an overlap condition requiring disengagement (Pierrot-Deseilligny et al., 1991).

Third, the SC is well known to be involved in saccadic eye movements. Clinical studies reported that children with autism made more frequent saccades during presentation of visual stimuli and in-between stimulus presentations (Kemner et al., 1998), and that inaccurate or slow saccadic movements were often observed in children/infants with autism (Rosenhall et al., 1988; Pensiero et al., 2009). These symptoms may be the result of abnormal activity of the SC or other brainstem areas related to eye movements in autism.

Fourth, the SC is an important structure for sensory gating. Prepulse inhibition (PPI) is an operational measure of sensorimotor gating in which a weak auditory prepulse attenuates the subsequent behavioral responses to a loud startling noise (Braff and Geyer, 1990). Human behavioral studies reported that patients with autism exhibited significantly less PPI (McAlonan et al., 2002; Perry et al., 2007), while there was a downward tendency of PPI in SC-lesioned monkeys (Saletti et al., 2014). In murine models of autism by prenatal exposure to valproic acid or genetic modification, deficits in PPI as well as decreases in parvalbumin-positive neurons in the SC were reported (Dendrinos et al., 2011; Nguyen et al., 2011; Nakamura et al., 2015).

Fifth, clinical studies suggest that dysfunctional serotonin signaling might contribute to abnormal autistic behaviors (Scott and Deneris, 2005). The SC is reported to be involved in a serotonin release in the cortex; electrical stimulation of the SC increased serotonin release in the frontal cortex (Dringenberg et al., 2003). This finding suggests that malfunctioning of the SC could induce a decrease in serotonin release in the cortex, which might induce autistic symptoms.

Sixth, patients with autism, and animals with exposure to valproic acid, show deficits in gamma oscillation in response to sensory stimulation (Gandal et al., 2010). Since PUL neurons show gamma oscillation in response to visual stimuli (Le et al., 2016), malfunctioning of the SC-PUL system could induce deficits in cortical gamma oscillation.

Finally, human morphological studies using MRI reported alteration in the amygdala and thalamus, including the pulvinar, in autism (Tsatsanis et al., 2003; Amaral et al., 2008; Tamura et al., 2010). Although no morphological alterations specific to the SC of patients with autism have been reported, fMRI anatomical comparisons indicate that significant differences in these patients occur in the whole midbrain (including the SC—smaller midbrain) (Brambilla et al., 2003).

Taken together, all of this evidence suggests the involvement of the SC-PUL pathway in ASD. The malfunction of the SC-PUL pathway in the early developmental stage might trigger developmental deficits in the other brain systems, including the cortical system. To our knowledge, this pathway has not been systematically investigated in the context of ASD but the SC and PUL are clearly compelling targets for the behavioral, motor, sensory, and attentional deficits observed in these disorders. Future studies could benefit from incorporating this perspective and examine more directly the role of SC-PUL in ASD.

## Author contributions

SCS and RSM organized the structure of the review and wrote the first draft, with the contribution of LAI. All the authors listed have made substantial, direct and intelectual contribution to the work and approved it for publication.

## Funding

This research was supported partly by Grant-in-Aid for Scientific Research (B) (16H04652) from Japan Society for Promotion of Science (JSPS), Japan, and by FEDER through the operation POCI-01-0145-FEDER-007746 funded by the Programa Operacional Competitividade e Internacionalização—COMPETE2020 and by National Funds through FCT—Fundação para a Ciência e a Tecnologia within CINTESIS, R&D Unit.

### Conflict of interest statement

The authors declare that the research was conducted in the absence of any commercial or financial relationships that could be construed as a potential conflict of interest.
